# A giant calculus within urethral diverticulum causing urinary retention in an uncommunicative woman

**DOI:** 10.1002/iju5.12435

**Published:** 2022-03-18

**Authors:** Takao Kawai, Yoshinosuke Amemoto

**Affiliations:** ^1^ Department of Urology Minami Seikyo General Hospital Nagoya Aichi Japan

**Keywords:** lithotripsy, urethral diverticulum, urinary calculi, urinary retention, urinary tract infections

## Abstract

**Introduction:**

There is no prior case report of calculus within a female urethral diverticulum causing urinary retention. We present such a case successfully treated by transurethral lithotripsy.

**Case presentation:**

A 34‐year‐old bedridden and uncommunicative woman with spinocerebellar degeneration presented with fever for 5 days. She was admitted to the hospital for a urinary tract infection with a 3‐cm calculus in the lower urinary tract. At the time of admission, acute urinary retention occurred. A bladder catheter was placed, and antibiotics were administered; both improved the urinary tract infection. Subsequently, transurethral lithotripsy was performed and revealed that the giant calculus was incarcerated within the urethral diverticulum. The bladder catheter was removed postoperatively, and urinary retention did not recur. No calculus reformation or urinary tract infections were observed for 6 months after discharge.

**Conclusion:**

A giant calculus within a urethral diverticulum may cause acute urinary retention in an uncommunicative patient.

**Abbreviations & Acronyms**
CT: computed tomography
UTI: urinary tract infection
WBC: white blood cell

**Keynote message**

A calculus within a urethral diverticulum is quite rare in women, and no case reports thus far have discussed the relationship between this condition and urinary retention. We report on a case of giant calculus within urethral diverticulum causing urinary retention, successfully treated by transurethral lithotripsy. An uncommunicative female patient with a calculus within a urethral diverticulum cannot report symptoms of dysuria, and this may result in acute urinary retention.

## Introduction

Urethral diverticulum is a rare condition in women, with a prevalence in adult women of 0.6–4.7%.^1^ Among the patients with urethral diverticulum, stone formation within the diverticulum occurs in only approximately 1.5–10%.^2^ A prior case report revealed that a calculus within a female urethral diverticulum causes prolonged lower urinary tract symptoms such as dysuria.^3^ Others have revealed that a large urethral diverticulum or advanced urethral diverticular adenocarcinoma can be the cause of urinary retention.^4^, ^5^ However, no report has described a calculus within the female urethral diverticulum, as a cause of acute urinary retention. We encountered the case of a bedridden and uncommunicative woman who had acute urinary retention due to a calculus within a urethral diverticulum, successfully treated by transurethral lithotripsy.

## Case presentation

**Fig. 1:**
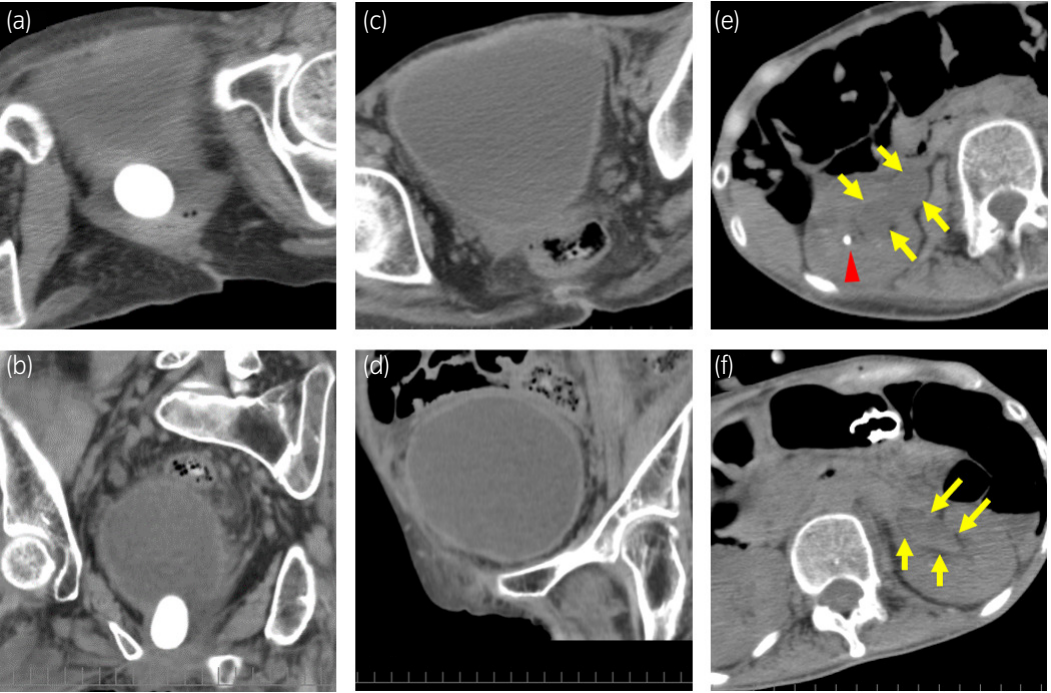
CT performed on the first day of hospitalization. (a,b) The giant calculus with a diameter of 3 cm was located on the dorsal and caudal side of the bladder. (c,d) A distended bladder was observed, and estimated volume of the bladder was about 690 mL (1/6 × 3.14 × 10 × 11 × 12). (e,f) The arrows showed bilateral hydronephrosis. The triangle indicated kidney stone.

**Fig. 2:**
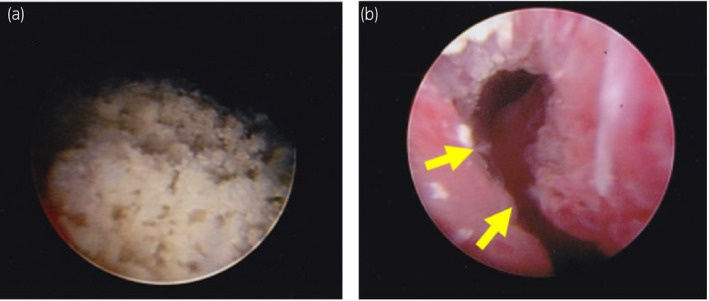
(a) Intraoperative images showed the rough and fragile calculus within the urethral diverticulum. (b) After extraction of calculus, the cavity of urethral diverticulum was observed. The arrows showed the orifice of urethral diverticulum.

**Fig. 3:**
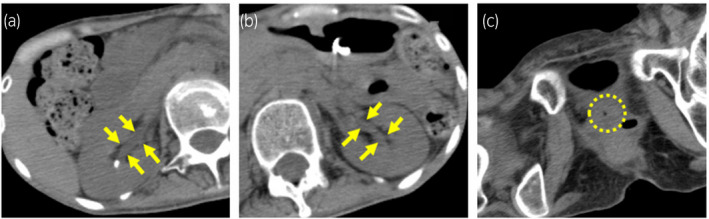
CT performed 6 months after the operation. (a,b) The arrows revealed that bilateral hydronephrosis disappeared. (c) The circle revealed that calculus was not detected at the same slice as Figure 1a.

A 34‐year‐old bedridden woman with spinocerebellar degeneration presented with fever (38°C) for 5 days. Her surgical history included a simple total hysterectomy and bilateral salpingo‐oophorectomy for an ovarian benign tumor 2 years prior; no complications such as dysuria were noted. Prior to admission, she was administered antibiotics (ceftriaxone) in a nursing home for 4 days. However, her fever did not improve and she was therefore admitted to the hospital. Her blood test showed elevated WBC (22 000/μL) and C‐reactive protein levels (8.73 mg/dL), whereas her serum creatinine concentration was normal (0.44 mg/dL). Urinalysis showed many urinary WBCs and negative for nitrite and bacteria. Chest CT did not reveal an active pneumonia infection. Abdominal CT revealed a 3 cm‐calculus in the lower urinary tract (Fig. 1a,b). Based on these results, the patient was diagnosed with UTI and was administered broad‐spectrum antibiotics (meropenem) because continuous high fever might imply the ineffectiveness of former antibiotics. Blood and urine cultures were found to be negative few days later, possibly due to antibiotic administration prior to culture sample collection. No information regarding the patient’s urinary output in the nursing home was available, and she was unable to communicate the need to urinate. However, after hospital admission, the patient’s urine output was poor, and both abdominal CT and ultrasonography revealed a distended bladder and bilateral hydronephrosis (Fig. 1c–f). Thus, the patient was diagnosed as having urinary retention. Following the diagnosis, a bladder catheter was inserted, and approximately 600 cc of urine was immediately voided. Her fever resolved on the 5th day of hospitalization, and her blood test results improved. On the 10th day of hospitalization, transurethral lithotripsy was performed to relieve urinary obstruction, using rigid nephroscope (diameter: 8mm, a viewing angle: 20°). The giant calculus could not be located within the urethra or bladder, until we finally localized the calculus incarcerated within a urethral diverticulum. We then fragmented and extracted the stone using lithotripter (Fig. 2). Laboratory analysis revealed the calculus was composed of magnesium ammonium phosphate (struvite). There were no postoperative complications, and removal of the bladder catheter 2 days after the operation did not cause recurrence of acute urinary retention. Diverticulectomy was deferred, as the patient guardian didn’t consent to the operation. No pyuria was observed at the 3‐ and 6‐months follow‐up after surgery and the post‐operative ultrasonography revealed the disappearance of distended bladder and bilateral hydronephrosis and no residual urine at the same follow‐up. The CT taken at the 6‐months follow‐up revealed that calculus within urethral diverticulum was not detected and bilateral hydronephrosis disappeared (Fig. 3). We will follow her every half year in the hospital for her whole life and check her by urinalysis, ultrasonography and CT.

## Discussion

The causes of urinary retention in women are divided into two categories: impaired detrusor activity in the bladder and bladder outlet obstruction.^6^ Common causes of detrusor underactivity include aging, diabetes, neurological diseases, medications, and even uterine leiomyomas.^7^, ^8^ Bladder outlet obstruction is commonly due to pelvic organ prolapse, iatrogenic obstruction, tumor, or dysfunctional voiding.^7^ In this case, the patient had some risk factors for urinary retention, such as spinocerebellar degeneration and surgical history. Unfortunately, urodynamic tests could not be performed because the guardian didn’t agree with the tests. However, the improvement of urinary retention after lithotripsy made us speculate that the cause of outlet obstruction in the patient was due to the presence of calculus in urethral diverticulum.

A stone analysis was performed, revealing the presence of struvite and proved that the calculus was created by chronic or recurrent UTI, which was consistent with the presence of kidney stone (Fig. 1e). A history of UTI leads to the stagnation of purulent urine within the urethral diverticulum, leading to the formation of a calculus. If a giant calculus is created in the urethral diverticulum, patients usually present with symptoms of voiding dysfunction such as incomplete emptying of the bladder, slow urine stream or urinary hesitancy.^3^ However, in this case the patient could not communicate these symptoms, so a calculus in the urethral diverticulum was not detected before admission to the hospital. In addition, a new infection caused urinary tract edema due to inflammation, predisposing the patient to complete urinary retention.

Surgical treatment is required for a calculus within urethral diverticulum.^9^, ^10^ In this case, the big orifice of urethral diverticulum enabled the operator to insert lithotripter into the cavity of urethral diverticulum, resulting in successful treatment by transurethral lithotripsy. We could not perform diverticulectomy; therefore, we must follow the patient carefully.

## Conclusion

This is the first case report of calculus in a female urethral diverticulum causing acute urinary retention successfully treated by transurethral lithotripsy. Calculus in urethral diverticulum should be considered a cause of acute urinary retention especially in an uncommunicative woman.

## Author Contributions

Takao Kawai: Conceptualization; Project administration; Writing – original draft; Writing – review & editing. Yoshinosuke Amemoto: Conceptualization; Writing – review & editing.

## Conflict of interest

The authors declare no conflict of interest.

## Approval of the research protocol by an Institutional Reviewer Board

Not applicable.

## Informed consent

Informed consent was obtained from the guardian of this patient.

## Registry and the Registration No. of the study/trial

Not applicable.
